# Supplementary material for: Defying Expectations: Extended Lifespan and Age‐Related Telomere Dynamics in the Tropical Bat Species, Molossus molossus
Source: Ecol Evol. 2026 Aug 9;16(8):e74125. doi: 10.1002/ece3.74125 (PMC13453432; doi:10.1002/ece3.74125)
Supplement: Supplementary file 1 [file ECE3-16-e74125-s001.zip › README.docx]

There are two datasets associated with this paper:

- Lonergan_Capturedata.xlsx (detailed records of bat captures)
- Lonergan_Molossus_Telomeres_Eco&Evo.csv (relative telomere length (z-scores) with sample metadata)

Description of data columns below:

**Capture Data Sheet**

Site: Capture location

Year: Year of capture.

SampleID: Unique sample code assigned to each bat.

Indiv: PIT tag or band number (unique individual identifier; NA if not tagged).

Genus: Taxonomic genus of the bat (Molossus).

Species: Species identification (e.g., molossus, coibensis).

Sex (M/F): Sex of the individual (M = male, F = female).

Age Cohort: Life stage at capture (J = juvenile, SA = subadult, A = adult).

Age: Estimated minimum age at capture, with “+” indicating unknown upper bound (e.g., “1+” means at least one year old).

SolidAge: Numeric transformation of age used in models (e.g., “1+” coded as 1).

Weight: Body mass at capture (grams).

Forearm: Forearm length (mm).

**Telomere Data Sheet**

SampleID: Unique sample identifier (matches Capture Data Sheet).

Indiv: Individual PIT tag/band identifier.

AssayPlate: qPCR plate identifier for telomere assay.

DNAPlate: Extraction plate identifier for DNA sample.

Site: Capture location.

Year: Capture year.

Sex: Sex of the individual (M/F).

Age: Estimated minimum age at capture, with “+” indicating unknown upper bound (e.g., “1+” means at least one year old).

SolidAge: Numeric transformation of age used in models (e.g., “1+” coded as 1).

KnownAge: Indicates whether the age is precisely known (Y = yes, N = no).

TelomereLengthZ: Relative telomere length (z-scored).
